# On the Re-Creation of Protoribosome Analogues in the Lab

**DOI:** 10.3390/ijms25094960

**Published:** 2024-05-02

**Authors:** Ilana Agmon

**Affiliations:** 1 Schulich Faculty of Chemistry, Technion—Israel Institute of Technology, Haifa 3200003, Israel; chilana@technion.ac.il; 2 Fritz Haber Research Center for Molecular Dynamics, Hebrew University, Jerusalem 9190401, Israel

**Keywords:** dimeric protoribosome, origin of life, ribosome, symmetrical region

## Abstract

The evolution of the translation system is a fundamental issue in the quest for the origin of life. A feasible evolutionary scenario necessitates the autonomous emergence of a protoribosome capable of catalyzing the synthesis of the initial peptides. The peptidyl transferase center (PTC) region in the modern ribosomal large subunit is believed to retain a vestige of such a prebiotic non-coded protoribosome, which would have self-assembled from random RNA chains, catalyzed peptide bond formation between arbitrary amino acids, and produced short peptides. Recently, three research groups experimentally demonstrated that several distinct dimeric constructs of protoribosome analogues, derived predicated on the approximate 2-fold rotational symmetry inherent in the PTC region, possess the ability to spontaneously fold, dimerize, and catalyze the formation of peptide bonds and of short peptides. These dimers are examined, aiming at retrieving information concerned with the characteristics of a prebiotic protoribosome. The analysis suggests preconditions for the laboratory re-creation of credible protoribosome analogues, including the preference of a heterodimer protoribosome, contradicting the common belief in the precedence of homodimers. Additionally, it derives a dynamic process which possibly played a role in the spontaneous production of the first bio-catalyzed peptides in the prebiotic world.

## 1. Introduction

The hub of contemporary life is the translation system that converts the genetic information stored in DNA into polypeptides, which subsequently fold into proteins. The capability to present a feasible scenario for the autonomous emergence of a simplified version of the contemporary translation system is therefore imperative for maintaining life emergence via natural processes. The essence of translation, i.e., the synthesis of polypeptides, occurs within the ribosome, an intricate RNA–protein complex comprising two subunits; the large subunit hosts peptide bond formation and nascent chain elongation, while the small subunit houses the mRNA carrying the genetic code and the decoding center. Peptide bond formation takes place in the PTC, the active site of the large ribosomal subunit, which consists solely of RNA [1,2]. The precise positioning of the aminoacylated 3′ ends of the aminoacyl-tRNA (A-tRNA) and peptidyl-tRNA (P-tRNA) within the PTC enables positional catalysis, aligning the reacting amino acids in optimal proximity and stereochemistry for an efficient peptide bond formation [3]. The nucleophilic attack of the amino group of the amino acid bound to A-tRNA on the carbonyl carbon of the C-terminal residue in the peptide attached to P-tRNA results in peptide bond formation [1] and in the incorporation of a new amino acid into the growing peptide chain. 

**Figure 1:**
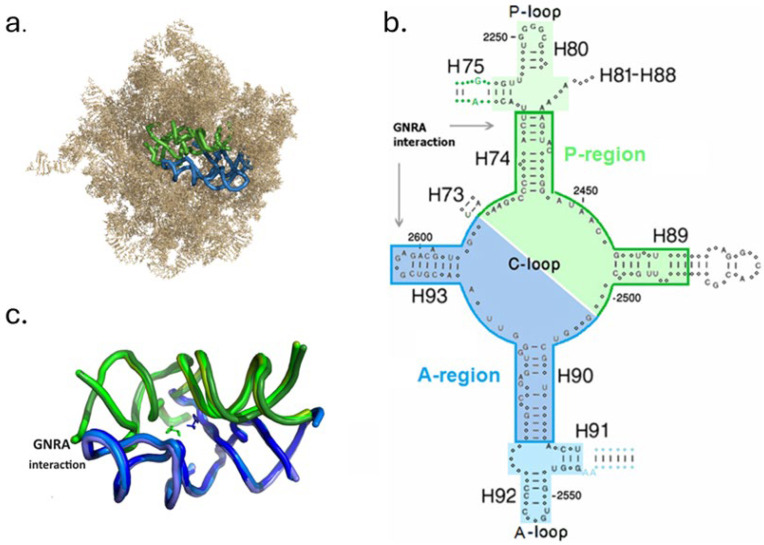
The protoribosome. (**a**) The symmetrical region encircling the PTC within the rRNA of the large subunit (pdb 2aw4). H68H71 were removed to divulge the PTC. A-region in blue hues, P-region in green hues, throughout. (**b**) A 2D scheme of the PTC neighborhood in symmetrical representation. The part assigned to the dimeric protoribosome (DPR) is framed and presented with stronger hues. The A- and P-loops, which make part of the SymR, are depicted by lighter background and the non-symmetrical extensions, H75 and H91, are indicated by blue and green letters and dots. Nucleotides conserved by more than 97% in each of the three domains of life are indicated by capital letters. (**c**) Overlap of the DPR pocket as found in archaea (pdb 1vq6), bacteria (pdb 2wdl), and eukarya (pdb 3u5d), portraying the extreme tertiary conservation of this region. The pocket is projected approximately along the symmetry axis and peptide bond is formed at the bottom of the cavity.

The contemporary ribosome, despite its general lack of internal symmetry, contains a symmetrical region (SymR) of about 180 nucleotides, which exhibits an approximate 2-fold rotational symmetry [4] (Figure 1a,b). This region encircles the PTC pocket (Figure 1c), whose walls are primarily formed by the nucleotides of the central loop of the domain V (C-loop). The symmetry pertains to the fold and nucleotide conformation of the two halves of the SymR, but not to the sequence. The universally conserved CCA tails (C74–A76) at the 3′ ends of the A- and P-tRNAs, along with their bound amino acids, are accommodated within the PTC with the 2-fold symmetry relating them as well [1]. C74 and C75 of P-tRNA form Watson–Crick base pairs with G2252 and G2251 (*E. coli* numbering throughout) in the loop of helix 80 (H80), referred to, accordingly, as P-loop, while C75 of the A-tRNA 3′ end base pairs with G2553 from the symmetric loop of H92, termed thus A-loop (Figure 1b). The SymR is highly conserved both structurally and sequentially across kingdoms [4], suggesting that it carries features predating the divergence into the life kingdoms. This region is believed to retain a remnant of a standalone primordial protoribosome [5,6,7,8], whose primacy in the ribosome evolution is supported by accretion models based on the distribution of A-minor motifs, insertion fingerprints, and helix elongation junctions in the rRNA of the large subunit [6,9].

The symmetry was interpreted as an indication that the initial protoribosome may have been a dimer of the two symmetrical monomers [5,6,7,8], which autonomously materialized in the prebiotic world through the spontaneous folding of the monomers and their dimerization. The sequences of monomers of 60–70 mer, as well as extended monomers of about 110 mer, derived from the PTC region of the contemporary bacterial and archaeal 23S rRNAs, have been shown through energy minimization using Mfold [10] to acquire 2D schemes akin to those found within the modern ribosome [8,11,12,13]. The materialization of these monomers would have depended on the existence of nucleotide chains of sufficient length. The generation of RNA strands of 100–300 mer on rock glasses [14] suggests that long RNA chains could have existed in the prebiotic environment, allowing the formation of the monomers. As RNA molecules tend to form stable dimers spontaneously [15], RNA dimers encompassing the PTC-ancestor pocket could have been readily available. The dimers would have catalyzed peptide bond formation between random amino acids and generate short peptides of random composition. 

The smallest and simplest model of a dimeric protoribosome analogue proposed so far is that of the DPR (Figure 1b,c), incorporating 121 nucleotides within the modern ribosome [8,16]. The DPR structure is assembled via the association of two symmetric L-shaped RNA monomers, designated as the A-DPR and P-DPR, comparable in size and shape to the tRNA [8,17]. The monomers, being part of the SymR, are related in fold and in nucleotide conformation, but their sequences do not conform to the symmetry. Within the ribosome, the RNA moieties interpreted as remnants of the DPR monomers are predominantly linked by a GNRA interaction, which is among the strongest forces that hold two RNA pieces together [18]. It forms an intricate network of hydrogen bonds on one end of the dimer (Figure 1b,c), supplemented by discrete hydrogen bonds distributed across the monomer’s binding interfaces [4,18]. 

Recently, three independent research groups have demonstrated the successful synthesis of peptide bonds and short peptides within dimeric constructs derived from the sequence of the PTC region of contemporary ribosomes [11,12,19]. Here the characteristics of these three dimers, presumed to emulate the initial protoribosome, are examined, aiming at shedding light on the structure and function of the protoribosome involved in the production of the earliest bio-catalyzed peptides in the prebiotic world. This insight may be useful for reconstructing an authentic protoribosome analogue in a laboratory setting.

## 2. Results

**Figure 2:**
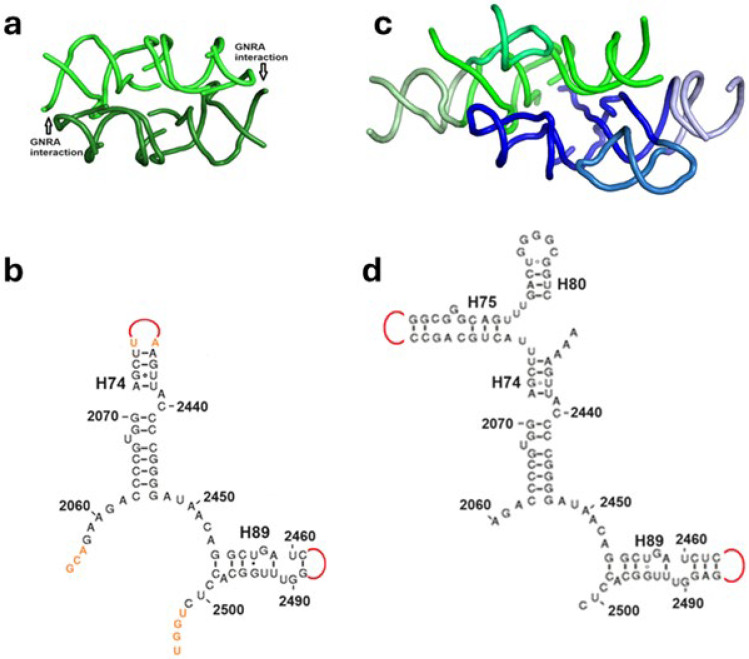
Fold and secondary structure of models I, II, and III, assumed to emulate the prebiotic protoribosome. (**a**) Structure of the P_I_P_I_’ and P_III_P_III_’ homodimers from models I and III (pdb 1vy4). GNRA loop derived from H93 is appended onto the truncated H89. A perpendicular view showing differences in the folds of P_I_ and P_III_ monomers is given in Figure S1. (**b**) A 2D scheme of the P_I_, P_III_ monomer sequences from Thermus thermophilus. Nucleotides common to both models are drawn in black and those existing solely in the P_III_-tailed monomer are shown in orange. The loops artificially capping the truncated helices are drawn in red. (**c**) Structure of the extended A_II_P_II_ heterodimer from model II. The DPR parts composing the PTC pocket are drawn in darker hues, the A-, P-loops in lighter hues, and the non-symmetrical extensions of H75 and H91 in lime and silver, respectively. (**d**) A 2D scheme of the P_II_ monomer sequence from Thermus thermophilus.

Three distinct dimeric structures, assumed to be associated with a standalone prebiotic protoribosome, were constructed [11,12,19], predicating on the approximate 2-fold rotational symmetry observed in the PTC region of the contemporary ribosome [4]. Only constructs that exhibited spontaneous folding, dimerization, and the capability to catalyze peptide bond formation, are considered as credible protoribosome analogues, and they are referred to as model I [12], model II [11], and model III [19]. Each of the dimeric models consists of constructs derived from the vicinity of the A- and P-regions of the modern ribosome (Figure 1b), designated accordingly as A- and P-monomers, which combine to form both heterodimers and homodimers. The heterodimer, AP, is assembled through the association of an A-monomer and a P-monomer around the symmetry axis, resulting in the formation of a cavity emulating the PTC. The homodimer AA’ is combined from an A-monomer and an A’-monomer, which is generated by rotating A by about 180° around the 2-fold symmetry axis to align optimally with its symmetrical P-monomer. The homodimer PP’ is similarly generated. The main structural characteristics of the three models are given in Figure 2 and in Table 1.

### 2.1. Structural Characterization of the Models

Model I [12] was selected from a series of 13 constructs derived from sequences of the PTC region in four bacteria. These constructs included homodimers composed of the A-DPR, P-DPR, A-DPR + A-loop, and P-DPR + P-loop (Figure 1b), which were tested for their ability to fold, dimerize, and catalyze the formation of a peptide bond. Among these, only four homodimeric constructs of the P-DPR monomers, P_I_P_I_’, were found to catalyze a peptide bond and they are represented by model I (Figure 2a,b, Table 1). Neither homodimers containing the A-, P-loops, nor A_I_A_I_’ homodimers of the A-DPR monomers, were found to catalyze the formation of the peptide bonds. Nevertheless, preliminary studies have shown that in some cases, A_I_P_I_ heterodimers synthesized peptide bonds. 

The sequence of the P_I_ monomer is identical to that of the P-DPR, except for the G2058A mutation at the 5′ nucleotide, intended to meet the requirement of T7 RNA polymerase. Additionally, H89 was shortened by two base pairs (Figure 2b, Table 1), allowing the GNRA loop artificially capping the truncated H89 to interact with the receptor base pairs in H74 (Figure 2a), analogously to the existing interaction of the GUGA loop of H93 with the H74 stem (Figure 1b,c). As both ends of the P_I_P_I_’ homodimer are designed to be held by this stabilizing interaction, the resulting dimer is expected to be rather rigid.

Model II [11] (Figure 2c,d) consists of homodimers, A_II_A_II_’, and heterodimers, A_II_P_II_ (Table 1). The homodimers are constructed from A-DPR, extended by the A-loop and by the non-symmetric helix H91, radiating from the SymR. Heterodimers combine the extended A-DPR with the extended P-DPR, i.e., with P-DPR augmented by the P-loop and by the non-symmetric H75 (Figure 2c,d). The non-symmetric extensions, H75 and H91, are located in the periphery of the PTC region, making no interactions with the inner walls of the PTC or with the reactants. Other alterations in the sequences of model II monomers, compared to that of the A-DPR and P-DPR, include the absence of nucleotides A2058 and A2059 in the P_II_ construct, which are the 5′ nucleotides of the P-DPR and of the entire region, and the absence of the 5′ nucleotide of the A-DPR, G2502, in the A_II_ construct. A_II_A_II_’ homodimers and A_II_P_II_ heterodimers were spontaneously folded and dimerized, generating RNA-K_9_ product, i.e., 9-mer poly-lysine bound to oligonucleotides of various lengths, but it remained unclear whether the homodimers or heterodimers carried out the catalysis.

The A_II_P_II_ heterodimers (Figure 2c) that emulate the PTC region are probably held by a GNRA interaction on one end, between the H93 loop and the receptor base pairs in the H74 stem. In A_II_A_II_’ homodimers, analogous GNRA interactions could have been formed, between the H93 loop and the stem of H90 (Figure 1b), if H90 possessed suitable base pairs. The participation of a GUGA loop in a GNRA interaction demands a CU:AG receptor sequence in the stem [20]. This configuration exists in the H74 stem, where C2073:G2436 and U2074:A2435 interact with the H93 GUGA loop (Figure 1b). However, the symmetrical nucleotides on H90 [4], A2516:U2568, and C2517:G2576, respectively, form the AC:GU sequence, which is not suitable for this type of interaction. In the absence of GNRA interactions, A_II_A_II_’ dimers would assemble via discrete hydrogen bonds, which could have led to a less stable protoribosome analogue. Such a dimer is possibly correlated with the FRET measurement that found a distance of 50 Å between the 3′ ends of the monomers assembling the dimer, whereas 23 Å was expected. This discrepancy was attributed by the authors to a “loose form dimer” [11], which is therefore unlikely to provide the reactants with the necessary proximity.

Model III [19] (Figure 2a,b and Figure S1) comprises two homodimeric versions: a basic model and a tailed model. Their sequences match that of the P-DPR (Figure 1b) and of its derived P_I_ monomer, except for alterations that include the addition of three nucleotides at the 5′ end (nucleotides 2055-57) and the elongation of the tip of H74 with the addition of U2075, A2434, which are not base paired in modern ribosomes. In the tailed model a segment of four nucleotides, UGGU, was added to C2501, the 3′ nucleotide of the basic model (Figure 2b, Table 1). The design of model III protoribosome analogue, same as that of model I, is expected to form dimers that are predominantly held by GNRA interactions on both sides (Figure 2a), being therefore rather rigid.

### 2.2. Functional Characterization of the Models 

In model I [12], stable P_I_P_I_’ dimers were formed and subsequently mixed with A-site and P-site substrate analogues. The product was synthesized via the formation of a single peptide bond, indicating a stationary process. Given that the monomer of model I is derived from the PTC without significant alterations, the substrates are hypothesized to access the dimer akin to the aminoacylated 3′ ends of A-, P-tRNAs, positioning the reactants at locations equivalent to the sites of the A- and P-amino acids in the PTC [12]. The absence of the A- and P-loops (Figure 1b and Figure 2a,b) in all the functionally active dimers of model I suggests that base pairs between C74, C75 (part of the P-substrate CCA-pcb) and the P-loop, as well as between C75 (from the A-substrate C-Pmn) and the A-loop, are not essential for the dimerization or peptide bond formation. The accommodation likely involves discrete hydrogen bonds between the substrates and PTC nucleotides that are within an interaction distance. 

In model II [11], the monomers were found to assemble homodimers, A_II_A_II_’, and heterodimers, A_II_P_II_. The distance between the two substrates accommodated in model II dimers, as measured using FRET, supports the assumption that the substrates bind to their dimer similarly to the way tRNAs bind to the ribosome [11], with the reactants located within the dimer akin to the amino acids in the PTC. Model II constructs contain the A- and P-loops, which can base pair with the 3′ CCA nucleotides of the ACCCACCA-K substrate. Nevertheless, as observed in the model I study [12], such base pairing could be unnecessary. The RNA moiety of the substrate is single-stranded, allowing flexibility and free rotation around the backbone bonds, which might impede base pairing. Substrate’s accommodation without base pairing can promote processivity by facilitating the release of deacylated substrates.

Model II dimers, A_II_A_II_’, A_II_P_II_, or both, were reported to spontaneously generate 9-mer poly-lysine products, raising a question concerned with the type of process that could have enabled processivity. An unbalanced number of A- to P-region contacts between the left and right sides of the PTC region was suggested to be linked with the dynamics of the PTC [4] and hypothesized to facilitate an early polymerization cycle [18]. The PTC region exhibits a GNRA network of hydrogen bonds on the far left-hand side of the dimer (Figure 1b,c), and only discrete hydrogen bonds, formed by flexible bulged nucleotides that combine and dissociate easily, on the right-hand side, between the symmetry-related H89 and H90 [18]. This spatial organization might be suggestive of an opening and closing mobility on one side of the dimer, possibly utilizing energy released by the peptide bond formation reaction to tear weaker hydrogen bonds. The unbalanced configuration of the A- to P-contacts may date back to the protoribosome, possibly being a remnant of an opening and closing mobility in the prebiotic PTC, which would have facilitated the release of deacylated substrates and the entry of the acylated ones.

Adopting this dynamic mechanism allows us to draw a hypothetical course of events that could have possibly taken place in model II dimers, as well as in the protoribosome. It suggests that after peptide bond formation, the growing peptide (di-peptide in the first round) would remain attached to the amino acid that performed the nucleophilic attack, akin to the process in the modern ribosome, while the deacetylated substrate would exit. Leaving would be possibly facilitated by an opening-closing mobility of the dimer and by substrate accommodation involving merely discrete hydrogen bonds. Subsequently, the vacant site would inhabit a new aminoacylated substrate that would perform the next nucleophilic attack, suggesting that the roles of the symmetric monomers, at this early stage of evolution, were not yet specialized as aminoacyl and peptidyl, in contrast to the modern ribosome [12]. 

**Figure 3:**
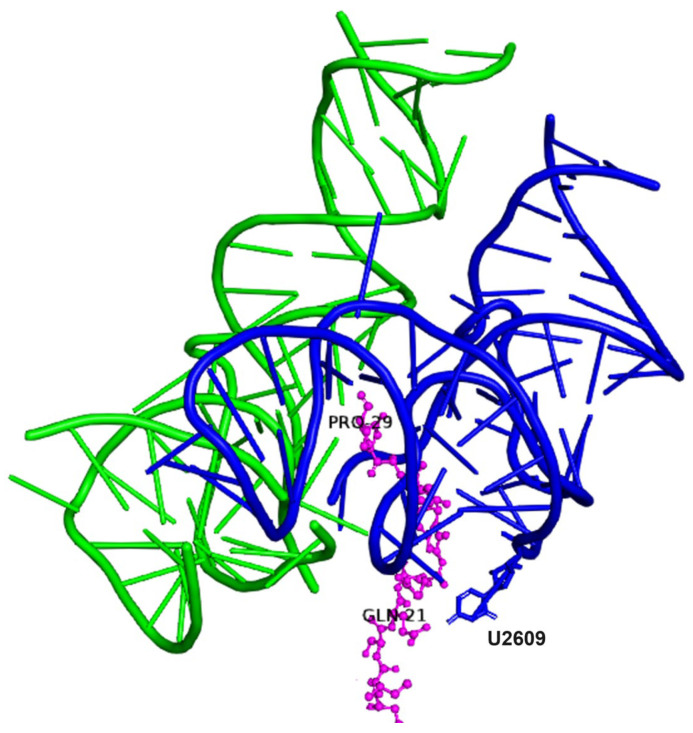
SecM peptide (magenta) in the PTC (pdb 3jbv). The nine C-terminal residues (Gln21-Pro29) interact with the PTC nucleotides included in model II.

This process is suggested to have been reiterated in model II dimers until the length of the product reached nine residues, but not beyond; a length that can be correlated with the number of amino acids protected by the protoribosome analogue. The crystal structures of the bacterial ribosomes bearing peptides, such as SecM (pdb 3jbv) and TnaC (pdb 4uy8), demonstrate that only the nine C-terminal residues of the nascent peptide interact with the nucleotides composing model II. The elongation of the product would stall when the N terminal residue reaches nucleotide U2609 in the bottom of the PTC (Figure 3). Incorporating the P-DPR 5′ nucleotides, A2058 and A2059 (Figure 1b), which are absent in P_II_ monomers (Figure 2d) and are located deeper in the PTC than U2609, could have perhaps resulted in the generation of 10-mer products by the heterodimers.

Model III [19] homodimers, P_III_P_III_’, present two modes of action, both static, i.e., synthesizing a single peptide bond. The basic dimer, exhibiting a relatively low level of peptide bond formation, appears to follow a similar pathway to that proposed for models I and II, that is, a process akin to peptide bond formation in the modern PTC.

The tailed dimer III demonstrated significantly higher productivity in peptide bond synthesis compared to the basic dimer III, and operated through a distinct mode of action. According to the authors’ suggestion [19], peptide bond synthesis could be attributed to the formation of four base pairs between the aminoacylated ACCA 3′ end of the minihelix substrate and the complementary UGGU tail, artificially added to the 3′ end of the P_III_ monomer, i.e., to C2501 (Figure 2b). The dimerization of the monomers, each forming these four base pairs with the substrate, is assumed to bring the two amino acids to proximity that allows peptide bond formation. And indeed, simulating the proposed system (File S1) confirms that the reactants attached to the P_III_ and P_III_’ monomers can be positioned close enough to allow peptide bond formation. However, in this layout, the substrates approach the PTC from the direction of the modern tunnel, i.e., from a direction opposite to that used by the substrates of the ribosome and likely by models I, II, and the basic model III. Moreover, the proximity of the reactants to nucleotide C2501 places them far from the accommodation sites of the reactants in the PTC. The distance according to the simulation is more than 20 Å, suggesting that peptide bond formation does not occur in this case akin to the contemporary ribosome. Furthermore, processivity would necessitate the detachment of at least four base pairs after each peptide bond formation to enable the release of the deacetylated substrate, and the absence of a systematic external energy source in the prebiotic era may present a challenge for the disruption of the bonds. The conditions for peptide bond formation are not highly specific [21], suggesting that the tailed P_III_P_III_’ dimer could have catalyzed the peptide bond by positioning the reactants with the required proximity, as observed previously in various systems [21,22,23,24,25], but its linkage to the protoribosome seems unlikely.

## 3. Discussion

### 3.1. Assessing Protoribosome Analogues

Under the assumption that the modern PTC still retains the necessary information for reconstructing an analogue of the ancient protoribosome capable of catalyzing peptide bond formation, several dimeric RNA entities were derived from the structure of the modern PTC region and tested for their ability to mediate peptide bond formation [11,12,19]. Models presented in the three studies are considered here as proper protoribosome analogues if they meet the following criteria:

1. The monomer sequence should be capable of folding spontaneously into a 2D scheme akin to the corresponding part of the PTC region.
2. The monomer should dimerize.
3. The dimer should catalyze the formation of a peptide bond.

Models I, II, and III satisfy criteria 1–3; that is, they can spontaneously fold into a 2D scheme analogous to that found in the PTC region [8,11,12,13,16], dimerize, and catalyze peptide bond formation [11,12,19].

4. The monomer’s sequence should have a realistic probability of accidentally occurring among the random RNA chains of the required length, while retaining the structural and functional characteristics of the modern PTC.

Only models I and III meet the fourth criterion, i.e., sequences of 60–70 mer, which preserve the structural and functional features of the modern PTC, were demonstrated to have a reasonable probability of occurrence in a pool of random RNA strands of that length [16]. However, the 110-mer sequences of the model II monomers are too long to meet such a requirement [16], suggesting that while model II may represent an evolved protoribosome, it is unlikely to have been the initial one. A recent study [26], reporting the formation of a PTC-mimic structure assembled from a 284-mer construct, is excluded from the current survey on the grounds of criteria 3 and 4. This RNA entity did exhibit structural similarity to the corresponding 2D and 3D structures of the PTC region and a capability of binding the A- and P-site substrate-analogues. However, peptide bond was not formed, and the probability of an accidental occurrence, in a primordial pool, of a sequence of such length that retains the distinct characteristics of the designed construct, is close to none.

5. To ensure evolutionary continuity, the process inferred from the dimer structure should be equivalent to that taking place in the modern ribosome. This entails the accommodation of the reactants akin to the positioning of the amino acids in the PTC, thereby preserving the current mechanism of the peptide bond formation.

The fifth criterion is likely to be met by models I, II, and the basic model III, which apparently conserve the accommodation configuration of the modern reactants [11,12] and can therefore be envisioned to have continuously evolved into the modern PTC. The tailed model III, however, which employs a distinct reaction site and mode of action compared to the modern PTC (File S1), is unlikely to have this capacity.

### 3.2. Heterodimer or Homodimer?

In the studies presenting models I and III, peptide bonds were predominantly obtained in PP’ homodimers, while in the study introducing model II, it remains uncertain whether the functional dimer was AA’, AP, or both. In all three studies, homodimers were assumed to constitute the initial protoribosome, which was suggested to materialize via duplication followed by dimerization [12,13,27,28]. A heterodimer was tested in the model II study [11], but its functionality was not determined, while preliminary results in the model I study [12] have shown that in some cases, heterodimers catalyzed peptide bond formation. This, and the fact that the contemporary peptide bond formation is catalyzed by a PTC region of heterodimeric nature (Figure 1b), imply that the preference of homodimers over heterodimers as the initial protoribosome is not straight forward. Furthermore, several conceptual considerations seem to favor a heterodimer as the initial protoribosome.

#### 3.2.1. Stereochemistry

In the pre-attack state of the ribosome, optimal configuration for the peptide bond formation necessitates that N, performing the nucleophilic attack, be positioned facing the carbonyl carbon target. A homodimer would fail to achieve this precise configuration because the symmetry would position the attacking N of the two reactants at the same height. The deviation from the optimal stereochemistry caused by symmetry is not severe, as evident from the experimental results obtained in the three studies, but a heterodimer, having only an approximate symmetry, can provide the optimal stereochemistry observed in the modern PTC. 

#### 3.2.2. Dynamics

If indeed the mobility of the dimers facilitated the entrance and release of the substrates, GNRA interactions on both sides of the dimer, as probably existed in the PP’ dimers of models I and III [12,19], could have led to a rigid entity, having a restricting effect on the mobility of the substrates. The A_II_A_II_’ dimer does not involve GNRA interactions due to the absence of suitable base pairs in H90. The assemblage of the dimer would occur via discrete hydrogen bonds that may result in a less stable dimer, possibly matching the “loose form” observed in the model II study [11], which is unlikely to provide the reactants with the necessary proximity. A heterodimer, on the other hand, offers both advantages; integrity is ensured through the GNRA interaction on one end (Figure 1b,c) and enhanced dynamics can be obtained via an opening-closing mobility of the other end of the dimer.

#### 3.2.3. Sequence

The monomers in a homodimer are expected to possess identical sequences. If the current PTC sequence had evolved from a homodimeric protoribosome, it would be expected, considering the high sequence conservation of the PTC region, to display at least some traces of the original symmetry between the monomer sequences. However, not only is such sequence symmetry absent in the current PTC, but the highly conserved A- and P- halves of the C-loop (Figure 1b), forming the walls of the PTC cavity, exhibit complementarity in bacteria [29], i.e., they are anti-symmetric.

#### 3.2.4. Origin of Life Perspective

The formation of a prebiotic homodimer necessitated the existence of two identical copies of the monomer sequence, which would self-assemble. The likelihood of an accidental occurrence of two identical sequences of 60–70 mer, which would form the two monomers, is infinitesimal. Homodimerization thus implies the primacy of a replication mechanism of some sort, which could have generated a second copy of the initial monomer sequence. The advent of prebiotic replicating enzymes, e.g., proto-polymerases, could not have occurred prior to the emergence of a coded protoribosome that would synthesize them. A replicase made of RNA had a negligible probability of emerging, due to, among other reasons, its weak affinity for its substrates [30], and the implausibility of a situation where one replicase would have retained its structure and function while copying a second unfolded one [31]. Despite intensive efforts, a non-enzymatic replication mode, capable of copying a sequence of about 60–70 mer, has not been accomplished so far [32]. Postulating that a homodimeric protoribosome predated the heterodimer thus implies that the protoribosome would have emerged only after an effective replication mode was already functional, but such mode is still unknown.

The alternative hypothesis, suggesting that a heterodimeric protoribosome was the precursor, appears more plausible. Probabilistic and energetic considerations have shown that a reasonable number of sequences prone to correctly fold, dimerize, and conserve the identity of the functional nucleotides within the PTC could have been found in a prebiotic pool of random RNA chains of 60–70 mer [16]. Two such sequences could have spontaneously folded into L-shaped monomers of unrelated sequences and dimerized to form the first non-coded protoribosome. The subsequent emergence of a sufficiently efficient replication mode, possibly enzymatic, would have generated more sequences of the initial monomers, as well as of their complementary sequences, enabling the formation of various types of homodimers and heterodimers, some of which would have probably been functional. The structure of the modern PTC, and particularly the complementarity exhibited by the C-loop nucleotides [29], supports the notion that a heterodimeric protoribosome, which was assembled from one of the initial monomers and its complementary sequence, was favored by evolution. 

### 3.3. Emergence of an Active Prebiotic Protoribosome

Combining insight gathered from the three studies [11,12,19], suggests a course of events that could have enabled the spontaneous emergence of a simple RNA entity, capable of catalyzing the formation of a single peptide bond and allowing its elongation. This scheme is applicable to the emergence and activity of the primordial protoribosome, as well as to the design of protoribosome analogues in the lab.

- Dimer formation—a dimer, probably a minimal heterodimer composed of two autonomously folded L-shaped monomers of 60–70 mer each, which harbors two substrates, could have been formed either through the dimerization of two monomers, each accommodating a substrate, or via the dimerization of the RNA monomers followed by substrates accommodation.
- Substrates—aminoacylated RNA segments comprising one to three nucleotides would be sufficiently long to act as substrates in vitro, because the remaining nucleotides, in longer RNA stretches, have nothing to pair with in the minimal, DPR-like, dimer. Nevertheless, longer RNA segments might have been present in the prebiotic substrate, possibly because they were involved in the aminoacylation of the RNA moiety [31,33].
- Peptide bond formation—reactants would be accommodated in the dimer akin to the modern PTC, in the required proximity and stereochemistry. A nucleophilic attack of one reactant on the carbonyl carbon of the second one would result in peptide bond formation.
- Processivity would possibly take place, if following the formation of a peptide bond, the deacylated substrate would depart and the next acylated substrate inhabit, allowing the formation of the next peptide bond. The reiteration of this dynamic process could have led to the production of peptides with random composition, distinguished from prebiotic mineral-catalyzed peptides by being homochiral, owing to the preference of the PTC, and its predecessor, the protoribosome, for L-amino acids [34].

The results the three scrutinized studies demonstrate experimentally the self-assembly of a functional apparatus that was capable of catalyzing the formation of a peptide bond and inducing its elongation, from RNA strands that could have occurred randomly in the primordial environment. It thereby establishes the viability of a spontaneous emergence of a dimeric protoribosome, which could have synthesized the first bio-catalyzed peptides in the prebiotic world, substantiating the notion that simple versions of key components of “life as we know it” could have autonomously originated from the inanimate materials. 

## 4. Materials and Methods 

The simulation of the tailed model III [File S1] was carried out on the structure of 70S ribosome from *Thermus thermophilus* (pdb 1vy4) using the molecular graphics program COOT [35]. The superposition of RNA segments was performed using the CCP4-8.0 program lsqkab [36].

## Figures and Tables

**Table 1:** Structural details of models I, II, and III.

	Monomer	Model I	Model II	Model III
Term in Figure 1b	P	P-DPR	P-DPR + P-loop + H75	P-DPR
Original names	P	tt-P1 *; tt-P1c; sa-P1c; ef-P1c	ptc1a	P1c2; P1c2^UGGU^
Nucleotides included	P	2058–2074; 2435–2461 *; 2489–2501;	2060–2085; 2234–2258; 2432–2463; 2487–2501;	2055–2075; 2434–2461; 2489–2501; +UGGU
Term in Figure 1b	A		A-DPR + A-loop + H91	
Original names	A		ptc1b	
Nucleotides included	A		2503–2528; 2535–2610	
Loops added to truncated helix	P	H74: 5′-CUUCGG-3′ H89: 5′-CUUCGG-3′ or: 5′-GUGA-3′	H75: 5′-GAAGAA-3′ H89: 5′-GUGAG-3′	H74: 5′-UUCG-3′ H89: 5′-GUGA-3′
Loops added to truncated helix	A		H91: 5′-GAAGAA-3′	
Length ** (nucleotides)	P	71 or: 67	108	70; 74
Length ** (nucleotides)	A		111	
Dimerization type		Homodimer PP’ (P_I_P_I_’)	Homodimer AA’ (A_II_A_II_’) + heterodimer AP (A_II_P_II_)	Homodimer PP’ (P_III_P_III_’)
Substrate	P	CCA-pcb #	ACCCACCA-K	Ala-minihelix^Ala^
Substrate	A	C-Pmn ##	ACCCACCA-K	Ala-minihelix^Ala^
Product		C-Pmn-pcb	CCA-K_9_ to CCCACCA-K_9_	alanylalanine
Species		*Thermus thermophilus*, *Staphylococcus aureus*, *Enterococcus faecium*	*Thermus thermophilus*	*Deinococcus radiodurans*

* H89 in tt_P1 is longer than tt_P1c by one base pair, U2462:A2488. ** including artificially added loops. # CCA-phenyl alanine-caproic acid-biotin. ## C-Puromycin.

## Data Availability

Data sharing is not applicable.

## Supplementary Materials

The supporting information can be downloaded at: https://www.mdpi.com/article/10.3390/ijms25094960/s1.
