# Supplementary material for: On the Re-Creation of Protoribosome Analogues in the Lab
Source: Int J Mol Sci. 2024 May 2;25(9):4960. doi: 10.3390/ijms25094960 (PMC11084786; doi:10.3390/ijms25094960)
Supplement: Supplementary file 1 [file ijms-25-04960-s001.zip › File S1.pdf]

File S1: Simulation of the $P_{III}P_{III}'$ dimer.

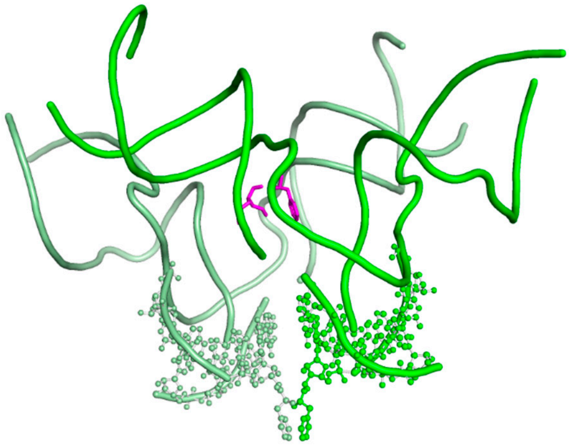

Figure S2: Simulation of the $P_{III}P_{III}'$ dimer [1] constructed from two tailed monomers (pdb 1vy4). Each tail is base paired to aa-ACCA of the hairpin substrate (in stick and ball representation). The reactants (at the bottom of the picture) are positioned in suitable proximity for peptide bond formation. The sites of the amino acids of the A-, P- tRNAs, located within the PTC, are shown in magenta.

Simulation procedure

The structure of the dimer $P_{III}P_{III}'$ [1] constructed from two tailed monomers, each carrying a reactant amino acid, was simulated in the following manner:

1. The structure of 70S ribosome from *Thermus thermophilus* (pdb 1vy4), determined at 2.6Å resolution, was used for the simulation.
2. The molecular graphics program WinCoot version 0.9.8.7 [2] was used for the simulation.
3. The 3′ end of P_{III} was elongated by one nucleotide, i.e., G2502 was added to C2501 (Figure 1b).
4. A helix of four base pairs, carrying an amino acid, was derived from the structure of an aminoacylated tRNA, by using only nucleotides 1-4:69-72, and attaching an amino acid to nucleotide 72 by overlapping aa-A76 onto nucleotide 72, using the CCP4-8.0 program lsqkab [3].
5. Nucleotide 1 of this helix was overlapped onto nucleotide G2502, forming a tail (Figure 2b, Figure S1) that elongated P_{III} by four nucleotides, 1-4, which are base paired to four nucleotides, 69-72, carrying an amino acid in its 3′ end, in agreement with [1, Figure 6].
6. The 5′ segment of P_{III}, containing the single stranded nucleotides 2055-2059, was removed to prevent bumping.

7. To allow proximity between the reactants, the entire aminoacylated helix was rotated around the bond joining nucleotide C2501 and G2502, which is capable of free rotation, until the amino acid reactant reached a proper position.
8. P_{III}′ monomer was generated by overlapping P_{III}, along with its attached aminoacylated helix, onto A_{III}, using lsqkab.
9. Dimerization indicated that the proximity between the amino acids (bottom of the Figure) is suitable for peptide bond formation.

References:

1. Kawabata, M.; Kawashima, K.; Mutsuro-Aoki, H.; Ando, T.; Umehara, T.; Tamura, K. Peptide bond formation between aminoacyl-minihelices by a scaffold derived from the peptidyl transferase center, *Life* **2022**, 12, 573.
2. Emsley, P.; Cowtan, K. Coot: model-building tools for molecular graphics. *Acta crystallographica section D: biological crystallography* **2004**, *60*, 2126-2132.
3. Kabsch, W. A solution for the best rotation to relate two sets of vectors. *Acta Crystallographica Section A: Crystal Physics, Diffraction, Theoretical and General Crystallography* **1976**, *32*, 922-923.
