# Supplementary figures and images for: On the Re-Creation of Protoribosome Analogues in the Lab
Source: Int J Mol Sci. 2024 May 2;25(9):4960. doi: 10.3390/ijms25094960 (PMC11084786; doi:10.3390/ijms25094960)

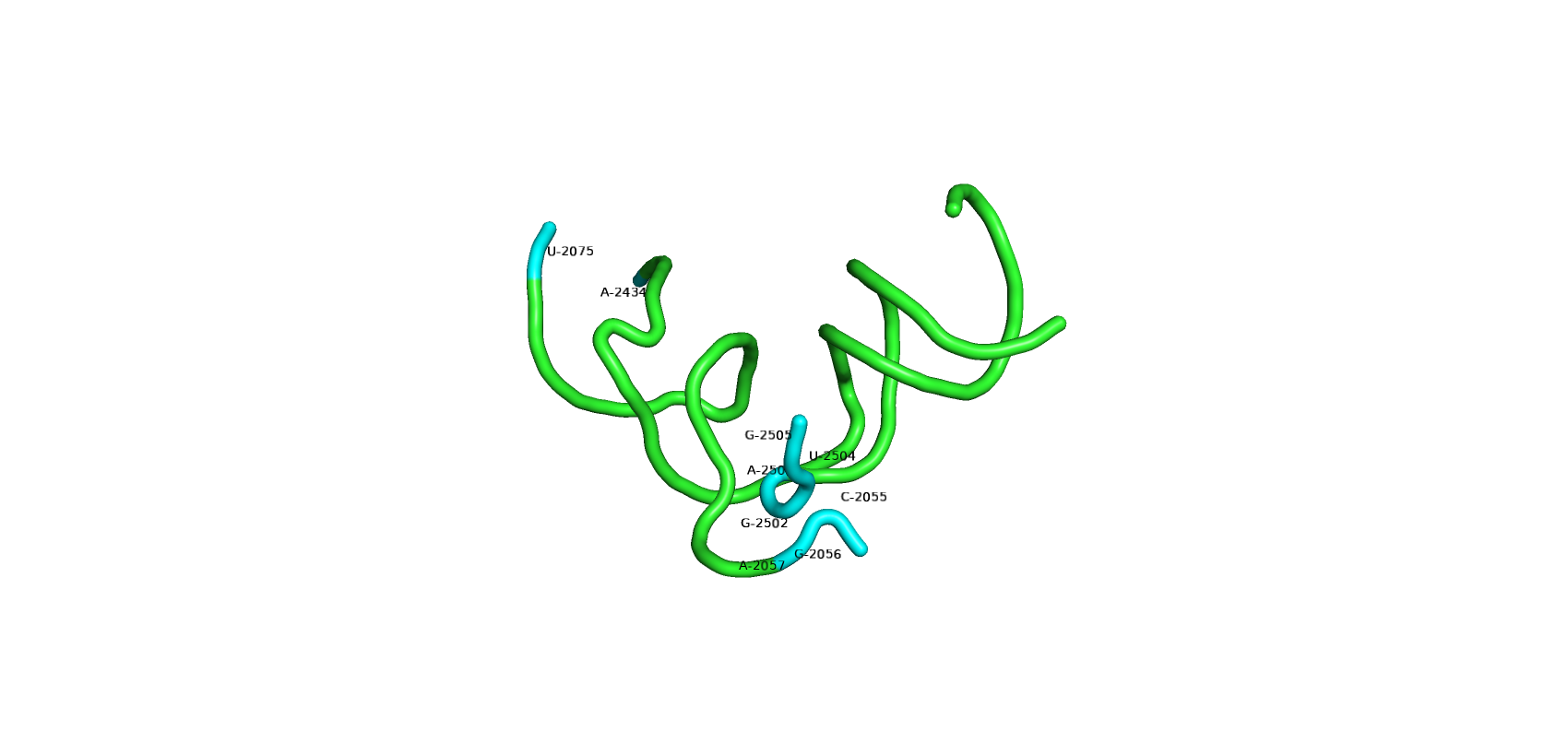

Supplement: Supplementary file 1 [file ijms-25-04960-s001.zip › Figure S1.png]
